# Long-term high-fat-diet feeding induces skeletal muscle mitochondrial biogenesis in rats in a sex-dependent and muscle-type specific manner

**DOI:** 10.1186/1743-7075-9-15

**Published:** 2012-02-21

**Authors:** Yolanda Gómez-Pérez, Gabriela Capllonch-Amer, Magdalena Gianotti, Isabel Lladó, Ana M Proenza

**Affiliations:** 1 Grup de Metabolisme Energètic i Nutrició, Departament de Biologia Fonamental i Ciències de la Salut, Institut Universitari d'Investigació en Ciències de la Salut (IUNICS), Universitat de les Illes Balears, Cra. Valldemossa km 7.5, E-07122, Palma de Mallorca, Spain; 2 Ciber Fisiopatología Obesidad y Nutrición (CB06/03), Instituto de Salud Carlos III, Spain

**Keywords:** Insulin sensitivity, Adiponectin, PGC-1α, TFAM, UCP3

## Abstract

**Background:**

Mitochondrial dysfunction is thought to play a crucial role in the etiology of insulin resistance, in which skeletal muscle is the main tissue contributor. Sex differences in skeletal muscle insulin and antioxidant responses to high-fat-diet (HFD) feeding have been described. The aim of this study was to elucidate whether there is a sex dimorphism in the effects of HFD feeding on skeletal muscle mitochondrial biogenesis and on the adiponectin signaling pathway, as well as the influence of the muscle type (oxidative or glycolytic).

**Methods:**

Gastrocnemius and soleus muscles of male and female Wistar rats of 2 months of age fed with a high-fat-diet (HFD) or a low fat diet for 26 weeks were used. Mitochondrial biogenesis and oxidative damage markers, oxidative capacity and antioxidant defences were analyzed. Serum insulin sensitivity parameters and the levels of proteins involved in adiponectin signaling pathway were also determined.

**Results:**

HFD feeding induced mitochondrial biogenesis in both sexes, but to a higher degree in male rats. Although HFD female rats showed greater antioxidant protection and maintained a better insulin sensitivity profile than their male counterparts, both sexes showed an impaired response to adiponectin, which was more evident in gastrocnemius muscle.

**Conclusions:**

We conclude that HFD rats may induce skeletal muscle mitochondrial biogenesis as an attempt to compensate the deleterious consequences of adiponectin and insulin resistance on oxidative metabolism, and that the effects of HFD feeding are sex-dependent and muscle-type specific.

## Background

Insulin resistance is a major risk factor for developing type 2 diabetes, which is caused by the inability of insulin-target tissues to respond properly to insulin [1], and in whose aetiology mitochondrial dysfunction is thought to play a crucial role [2,3]. Skeletal muscle is the main tissue responsible for the insulin-stimulated disposal of glucose and is the main contributor to the development of insulin resistance in type 2 diabetes [4].

Skeletal muscle is a heterogeneous tissue made up of different contractile fibre types, in which the relative importance of glycolysis and mitochondrial oxidative phosphorylation for energy production varies. Glycolytic muscles are mainly composed of fast twitch or fast glycolytic fibres and generate energy by means of anaerobic metabolic processes, whereas oxidative muscles have a high proportion of slow twitch or slow oxidative fibres, are very resistant to fatigue and obtain energy through oxidative metabolic processes [5,6]. Under normal feeding conditions, glycolytic muscles use mainly glucose metabolism, whereas oxidative muscles are highly dependent upon lipids [7]. Because of the differences between muscle types in energy demand and reliance on mitochondrial oxidative activity, differences in mitochondrial function can not be ruled out.

Skeletal muscle oxidative capacity is mainly determined by mitochondrial function and biogenesis [8]. Mitochondrial biogenesis involves both proliferation and differentiation processes, which imply an increase in mitochondrial content and an improvement of the functional capabilities of pre-existing mitochondria, respectively [9]. Mitochondrial biogenesis requires the coordinate participation of both mitochondrial and nuclear genomes [10] through numerous transcription factors. Peroxisome proliferator-activated receptor-γ coactivator-1α (PGC-1α) coactivates different transcription factors in response to energy requirements resulting in the activation of nuclear genes involved in mitochondrial biogenesis. Among them, mitochondrial transcription factor A (TFAM) is one of the regulatory factors needed for proper transcription of mitochondrial DNA and of the genes encoding subunits of respiratory complexes [11,12]. Mitochondrial dysfunction has been proposed to be involved in the alteration of oxidative metabolism associated to insulin resistance. However, the cause-and-effect relationship between mitochondrial dysfunction and the development of insulin resistance remains unclear [2,3,13].

High-fat diet feeding (HFD) leads to obesity and to an impairment of insulin sensitivity [14]. Women seem to be more protected from obesity-associated insulin resistance than men [15]. This protection has been attributed to the sex hormone milieu and has also been associated to differences in body fat distribution and adipokine levels. In this sense, women have been found to have significantly higher adiponectin plasma concentrations than men [15,16]. Adiponectin is a hormone secreted by adipocytes that circulates in high concentrations in serum and plays an important role in the regulation of mitochondrial biogenesis and insulin sensitivity [17,18]. Adiponectin binds to its receptors (AdipoR1, the most abundantly expressed in skeletal muscle, and AdipoR2) activating 5'-AMP-activated protein kinase (AMPK), which finally leads to the stimulation of glucose uptake and fatty acid oxidation. AMPK has also been implicated in the regulation of PGC-1α, the master regulator of mitochondrial biogenesis [17,18].

Sex differences have been previously described in mitochondrial biogenesis of skeletal muscle [19] and of other tissues, such as liver [20,21] brain [22], heart [23] and brown adipose tissue [24,25]. Moreover, we have also reported a higher skeletal muscle antioxidant capacity and a better insulin sensitivity profile in response to high fat diet (HFD) feeding in female rats compared to males [26]. Taking this background into account, the aim of the present study was to elucidate whether sex differences in the effects of HFD feeding on insulin sensitivity might be associated to differences in muscle mitochondrial biogenesis and the adiponectin signaling pathway, and whether these effects are dependent on muscle type.

## Methods and materials

### Animals and diets

Animal experiments were performed in accordance with the general guidelines approved by EU regulations (86/609/EEC and 2003/65/CE) and our institutional ethics committee. Male and female Wistar rats of 2 months of age (Charles River, Barcelona, Spain) were housed two per cage with free access to food and water and were kept at 22°C under a 12-hour light-dark cycle. Both male and female rats were divided into two groups (8-10 rats per group) with a similar body weight (333 ± 4 g for male rats and 215 ± 4 g for female rats) and were fed a low fat diet (3,385 Kcal/Kg diet; 2.9% fat by weight; A04, Panlab, Barcelona) or a high fat diet (HFD, 3,876 Kcal/Kg diet; 26% fat by weight) for 26 weeks. The HFD (namely cafeteria diet) components were cookies, pork liver pâté, fresh bacon, chocolate and ensaïmada (a typical Majorcan pastry) (Table 1). The energy composition of the HFD was 13% protein, 33% carbohydrate and 54% lipid, whereas the low fat diet (A04, Panlab, Barcelona, Spain) was 19% protein, 73% carbohydrate and 8% lipid. Animal body weights were assessed weekly and food intake fortnightly throughout the dietary treatment (animal final body weights were: 546 ± 9 g for control male rats, 675 ± 25 g for HFD male rats, 295 ± 7 g for control female rats and 462 ± 24 g for HFD female rats). All the components of the HFD were presented in several small pieces and in gross excess so as to allow the recovery the following day of at least part of all the components offered. The amount of each component consumed by each rat was calculated from the difference between the amount offered and the amount recovered the next day. Rats were sacrificed by decapitation after a 12-hour-period of fasting. Blood was collected and soleus and gastrocnemius skeletal muscles were rapidly dissected and weighed. Serum samples and a piece of each muscle were frozen in liquid N_2_and stored at -80°C until analyzed; the rest of the tissues were immediately processed. Pieces of muscle were homogenized at 4°C in a proportion of 1 g of muscle in 10 ml of buffer (50 mM HEPES, 100 mM NaF, 10 mM EDTA, 1 mM Na_3_VO_4_, 1% Triton X-100, 2 mM PMSF, 10 μg/ml aprotinin, 10 μg/ml leupeptin, pH 7.4).

**Table 1:** Food and nutrient composition of HFD intake

Nutrient composition (g/100 g food)				
**High fat food**	**Total food amount (g/100 g diet)**	**Protein**	**Carbohydrate**	**Lipid**

Fresh bacon	46.1 ± 2.7	17.3	-	29.9

Cookies	2.80 ± 0.89	5.82	68.0	21.3

Pork liver paté	26.1 ± 2.1	11.9	2.70	29.5

Ensaïmada	15.2 ± 1.9	8.10	50.6	29.1

Chocolate	4.35 ± 0.97	6.70	60.0	30.0

Pelleted standard diet	5.09 ± 1.67	18.7	73.3	8.00

Total food amount is the amount of each high fat food consumed expressed per 100 g of diet consumed. Given that no sex differences were found in high fat food consumption, the values of total food amount per 100 g of diet are the means ± SEM taking into account rats of both sexes (n = 12).

### Materials

Accutrend^® ^GCT-meter and glucose and triglyceride test strips were supplied by Roche Diagnostics (Basel, Switzerland). Enzyme immunoassay kits were used for measurement of rat serum insulin (Mercodia, Uppsala, Sweden), and total and high molecular weight adiponectin (Phoenix Pharmaceuticals Inc., Belmont, CA, USA and Biovendor, Heidelberg, Germany, respectively). The triglyceride measurement kit was acquired from Linear Chemicals SL (Barcelona, Spain). OxyblotTM Protein Oxidation Detection kit and antibodies to rat UCP3 (Cat. Num. AB4036) and PGC-1α (Cat. Num AB3242) were purchased from Chemicon International (Temecula, CA, USA). AdipoR1 (Cat. Num. ADIPOR12-A) and CPT1 (Cat. Num. CPT1M11-A) antibodies were from Alpha Diagnostic International (San Antonio, TX, USA). AMPKα (Cat. Num. 2532) and p-AMPKα (Cat. Num. 2531) antibodies were from Cell Signaling Technology (Danvers, MA, USA). TFAM antibody was kindly provided by Dr. H. Inagaki [27]. COXII antibody (Cat. Num sc-23984) was from Santa Cruz Biotechnology (Santa Cruz, CA, USA) and COXIV antibody (Cat. Num MS407) was from MitoSciences (Eugene, OR, USA). Mn-SOD (Cat. Num 574596) and Cu-SOD (Cat. Num 574597) antibodies were obtained from Calbiochem (San Diego, CA, USA). Chemiluminescence kit (ECL) for immunoblot development was purchased from BioRad (Hercules, CA, USA). High Pure PCR Template Preparation Kit and Oligonucleotide primer sequences were from Roche Diagnostics (Basel, Switzerland) and SYBR^® ^Green Quantitative RT-PCR kit was from Sigma-Aldrich (St. Louis, MO, USA). Routine chemicals used were supplied by Pronadisa (Madrid, Spain), Panreac (Barcelona, Spain) and Sigma-Aldrich (St. Louis, MO, USA).

### Serum glucose, insulin, adiponectin and triglyceride levels

Serum parameters were measured using the Accutrend^® ^system (glucose and triglyceride levels) and enzyme immunoassay kits (insulin and adiponectin levels). Homeostasis Model Assessment HOMA-IR was used to estimate insulin resistance [28] and was calculated as [fasting glucose (mM) × fasting insulin (μU/mL)]/22.5.

### Skeletal muscle composition

Total protein was determined in homogenates as previously described [29]. Triglycerides were measured spectrophotometrically in homogenates with a commercial kit.

### Measurements of skeletal muscle thiobarbituric acid-reactive substances (TBARS) and protein carbonyl groups

TBARS levels were measured as previously described [30] and used as an index of lipid peroxidation. Protein carbonyl groups were determined as index of protein oxidation by Dot-Blot detection using the OxyBlot™ Protein Oxidation Detection Kit according to the manufacturer's protocol with several modifications [22].

### Extraction and quantification of mitochondrial DNA

Mitochondrial DNA (mtDNA) was extracted by digestion of muscle homogenates with proteinase K (100 μg/μl) in a buffer containing 50 mM KCl, 10 mM Tris-HCl, 2.5 mM MgCl_2 _and 0.5% Tween 20. Mitochondrial samples were incubated overnight at 37°C and then boiled for 5 min. Mitochondrial DNA was linearised by digestion with Bcl I restriction enzyme for 3 h at 50°C and again boiled for 5 min. Samples were centrifuged at 7000 g for 5 min and the resulting supernatant was used for amplification. Real-time PCR was performed to amplify a 162-nt region of the mitochondrial NADH dehydrogenase subunit 4 gene. The primer sequences were 5'-TACACGATGAGGCAACCAAA-3' and 5'-GGTAGGGGGTGTGTTGTGAG-3'. The PCR product was purified with the High Pure PCR Template Preparation Kit and the concentration of the purified template was determined spectrophotometrically. Increasing amounts of template were amplified in parallel reactions to obtain a standard curve. Amplification was carried out in a LightCycler rapid thermal cycler system (Roche, Switzerland) using a total volume of 10 μl containing 0.375 μM of each primer, 3 mM MgCl_2_, 5 μl Master SYBR Green and 2.5 μl of sample. The PCR reactions were cycled 35 times after initial denaturation (94°C, 2 min), with the following parameters: denaturation at 94°C for 15 s, annealing and extension at 60°C for 1 min.

### Western blot analysis

Homogenized samples were centrifuged for 20 min at 13,000 × g and at 4°C and supernatants were collected as previously reported [31]. Fifty or 100 μg of soleus and gastrocnemius homogenate protein was fractionated on 8, 10 or 12% SDS-PAGE gels. At least one sample from each experimental group was loaded in each gel, and gels were run simultaneously. Protein charge was controlled by loading the same control sample in each gel. Gels were electrotransferred onto a nitrocellulose filter. Rabbit polyclonal antibodies to rat AdipoR1, AMPKα, p-AMPKα, UCP3, PGC-1α, TFAM and CPT1, goat polyclonal antibody against COXII and mouse monoclonal antibody against COXIV were used as primary antibodies. Development of immunoblots was performed using an enhanced chemiluminescence kit. Bands were visualized with the ChemiDoc XRS system (Bio-Rad, CA, USA) and analyzed with the image analysis program Quantity one^© ^(Bio-Rad, CA, USA). Bands revealed an apparent molecular mass of 18 kDa (COXIV), 25 kDa (TFAM), 27.5 kDa (COXII), 37 kDa (UCP3), 42 kDa (AdipoR1), 62 kDa (AMPKα and p-AMPKα), 88 kDa (CPT1) and 92 kDa (PGC-1α). Band density of each gel were corrected by band density of the control sample loaded in the same gel.

### Statistical analysis

All data are expressed as mean values ± SEM of 8-10 animals per group. Statistical analyses were performed using a statistical software package (SPSS 19.0 for Windows, Inc., Chicago, IL, USA). Statistical differences between experimental groups were analyzed by two-way analysis of variance (ANOVA) followed by Student's *t*-test as a post-hoc comparison. A p-value of less than 0.05 was considered statistically significant.

## Results

### Energy intake, biometrical parameters and skeletal muscle composition

Control female rats showed a higher energy intake and lower body weight gain than males (Table 2). HFD feeding increased energy intake and body weight in both sexes and the body weight increase was higher in female rats than in males. Gastrocnemius and soleus muscle weights (Table 3) were higher in male rats compared to females and increased with HFD feeding in female rats. Relative gastrocnemius weight was higher in control female rats than in their male counterparts and decreased with HFD feeding in both sexes. Non significant sex and HFD effects were found in the relative weight of soleus muscle. Soleus triglyceride content was higher in male rats than in females and increased with HFD feeding in female rats. Non significant sex and HFD effects were found in gastrocnemius triglyceride levels. Soleus protein content decreased in male rats with HFD feeding and their values were lower than those of their female counterparts. Non significant sex and HFD effects were found in gastrocnemius protein content.

**Table 2:** Energy intake and body weight gain

	*Control*	*HFD*	*ANOVA*
**Energy intake (Kcal/Kg day)**			
Male	169 ± 7	269 ± 11^a^	S, D
Female	294 ± 18^b^	332 ± 40	

**Body weight gain (%)**			
Male	64.1 ± 2.2	107 ± 7^a^	S*D
Female	40.0 ± 2.0^b^	112 ± 11^a^	

Body weight gain is calculated relative to body weight at the beginning of the treatment. Values are expressed as the mean ± S.E.M of eight animals per group. ANOVA (p < 0.05): S sex effect, D HFD effect, S*D sex and HFD interactive effect and NS non significant effect. Student's *t*-test (p < 0.05):^a ^HFD vs control, ^b ^female vs male.

**Table 3:** Skeletal muscle weight and composition

	*Gastrocnemius*			*Soleus*		
	*Control*	*HFD*	*ANOVA*	*Control*	*HFD*	*ANOVA*
**Tissue weight (g)**						
Male	5.14 ± 0.16	5.23 ± 0.08	S	0.244 ± 0.018	0.262 ± 0.024	S, D
Female	3.20 ± 0.12^b^	3.59 ± 0.11^a, b^		0.152 ± 0.018^b^	0.226 ± 0.014^a^	

**Relative tissue weight (g/Kg)**						
Male	9.27 ± 0.33	8.01 ± 0.29^a^	S, D, S*D	0.440 ± 0.035	0.401 ± 0.039	NS
Female	11.1 ± 0.4^b^	7.88 ± 0.37^a^		0.525 ± 0.065	0.497 ± 0.035	

**Triglyceride (mg/g tissue)**						
Male	6.88 ± 0.89	8.79 ± 1.77	NS	22.2 ± 3.2	19.1 ± 1.5	S, S*D
Female	7.78 ± 1.85	10.9 ± 1.3		12.5 ± 1.6^b^	18.7 ± 1.0^a^	

**Protein (mg/g tissue)**						
Male	81.6 ± 2.4	84.7 ± 3.8	NS	76.3 ± 1.9	60.8 ± 1.4^a^	D
Female	81.5 ± 1.8	78.7 ± 4.7		75.2 ± 4.0	69.8 ± 2.1^b^	

Values are expressed as the mean ± S.E.M of eight animals per group. ANOVA (p < 0.05): S sex effect, D HFD effect, S*D sex and HFD interactive effect and NS non significant effect. Student's *t*-test (p < 0.05):^a ^HFD vs control, ^b ^female vs male.

### Serum parameters

Serum insulin, triglyceride and total adiponectin levels and HOMA-IR values were higher in control male rats than in females (Table 4), but increased with HFD feeding only in female rats. Serum high molecular weight (HMW) adiponectin levels decreased with HFD feeding in male rats, and HFD female rats showed higher levels than HFD males. HMW adiponectin/total adiponectin ratio, which represents the proportion of the most active form of adiponectin for insulin sensitizing effects [18], decreased with HFD feeding only in males and, as a consequence, values of HFD male rats were lower than those of their female counterparts.

**Table 4:** Serum parameters

	*Control*	*HFD*	*ANOVA*
**Glucose (mM)**			
Male	6.76 ± 0.23	6.34 ± 0.20	NS
Female	5.95 ± 0.37	6.37 ± 0.36	

**Insulin (μg/L)**			
Male	1.79 ± 0.36	1.92 ± 0.47	S
Female	0.442 ± 0.124^b^	0.976 ± 0.151^a, b^	

**HOMA-IR**			
Male	14.6 ± 2.4	11.4 ± 2.2	S, S*D
Female	2.45 ± 0.64^b^	7.01 ± 0.70^a,b^	

**Total Adiponectin (ng/μL)**			
Male	5.86 ± 0.20	5.97 ± 0.46	D, S*D
Female	5.06 ± 0.39^b^	6.49 ± 0.31^a^	

**HMW Adiponectin (ng/μL)**			
Male	3.86 ± 0.53	2.39 ± 0.32^a^	S*D
Female	3.63 ± 0.36	4.11 ± 0.40^b^	

**HMW Adiponectin/Total Adiponectin**			
Male	0.655 ± 0.078	0.405 ± 0.054^a^	S, D
Female	0.773 ± 0.121	0.636 ± 0.053^b^	

**Triglyceride (g/L)**			
Male	2.58 ± 0.25	2.43 ± 0.20	D, S*D
Female	1.67 ± 0.14^b^	2.68 ± 0.21^a^	

HOMA-IR was calculated as [fasting glucose (mM) × fasting insulin (μU/mL)]/22.5. Values are expressed as the mean ± S.E.M of ten animals per group. ANOVA (p < 0.05): S sex effect, D HFD effect, S*D sex and HFD interactive effect and NS non significant effect. Student's *t*-test (p < 0.05):^a ^HFD vs control,^b ^female vs male.

### Gastrocnemius and soleus AdipoR1, AMPK and p-AMPK protein levels

**Figure 1:**
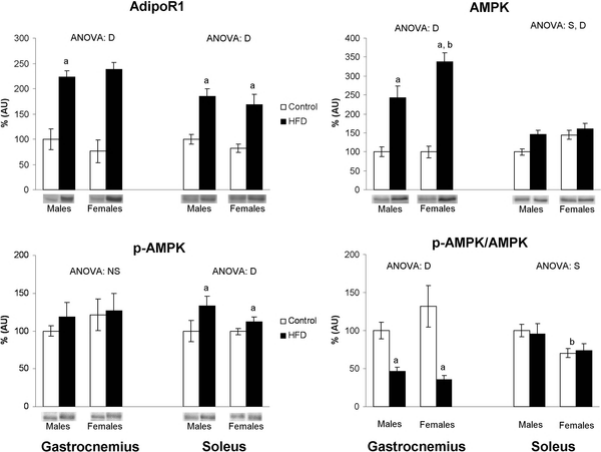
**Skeletal muscle AdipoR1, AMPK and p-AMPK protein levels**. Values of protein levels are expressed as % AU (arbitrary units), where control male rats were set as 100%. Data are expressed as the mean ± S.E.M of eight animals per group. ANOVA (p < 0.05): S sex effect, D HFD effect and NS non significant effect. Student's *t*-test (p < 0.05): ^a ^HFD vs control, ^b ^female vs male.

Gastrocnemius and soleus muscle AdipoR1 protein levels (Figure 1) increased with HFD feeding in both sexes, and no significant differences between sexes were found. In gastrocnemius muscle, HFD feeding increased AMPK levels, to a higher degree in female rats (237%) than in males (143%). The p-AMPK/AMPK ratio, which represents the proportion of active AMPK, decreased in both sexes (the decrease was 54% in male rats and 73% in females). No differences between sexes in these parameters were found in control rats. In soleus muscle, control male rats showed a higher p-AMPK/AMPK ratio than females. In response to HFD feeding, male rats increased AMPK and p-AMPK protein levels and maintained p-AMPK/AMPK ratio, which is lower in female rats, unaltered.

### Gastrocnemius and soleus muscles mitochondrial biogenesis markers

**Figure 2:**
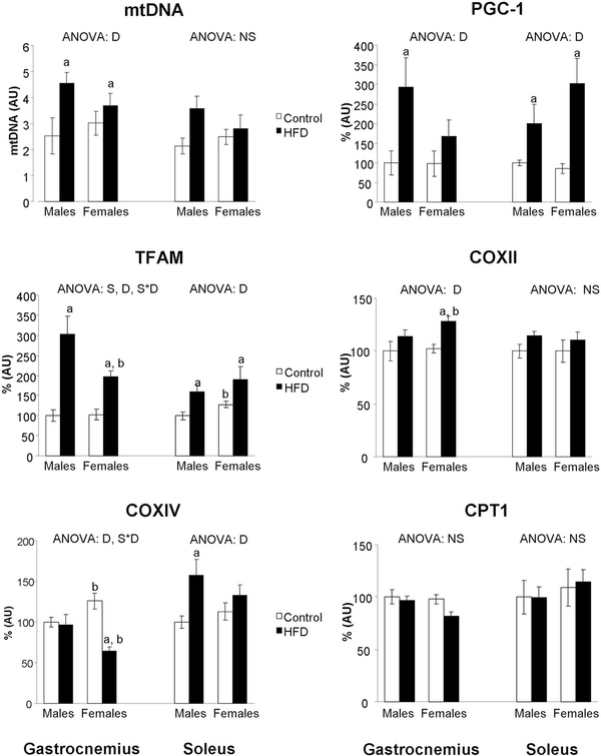
**Skeletal muscle PGC-1, TFAM, COXII, COXIV, CPT1 proteins and mitochondrial DNA levels**. Levels of mtDNA are expressed as AU (arbitrary units). Protein levels are expressed as % AU, where control male rats were set as 100%. Values are expressed as the mean ± S.E.M of eight animals per group. ANOVA (p < 0.05): S sex effect, D HFD effect, S*D sex and HFD interactive effect and NS non significant effect. Student's *t*-test (p < 0.05): ^a ^HFD vs control, ^b ^female vs male.

HFD feeding increased mtDNA levels in both sexes and in both muscles, although only reached statistical significance in gastrocnemius. In this muscle, control female rats showed higher COXIV protein than their male counterparts (Figure 2), whereas no differences between sexes were found in PGC-1α, TFAM or COXII protein levels. HFD feeding increased TFAM levels in both sexes, PGC-1α levels in male rats and COXII protein levels in female rats. In contrast, HFD feeding decreased COXIV protein levels in female rats.

In soleus of control rats, TFAM protein levels were higher in females than in males, but no differences between sexes were found in PGC-1α, COXII or COXIV protein levels. HFD feeding increased PGC-1α and TFAM levels in both sexes and COXIV levels in male rats. No statistically significant differences were found in both COXII and CPT1protein levels.

### Gastrocnemius and soleus oxidative damage and UCP3 levels

**Figure 3:**
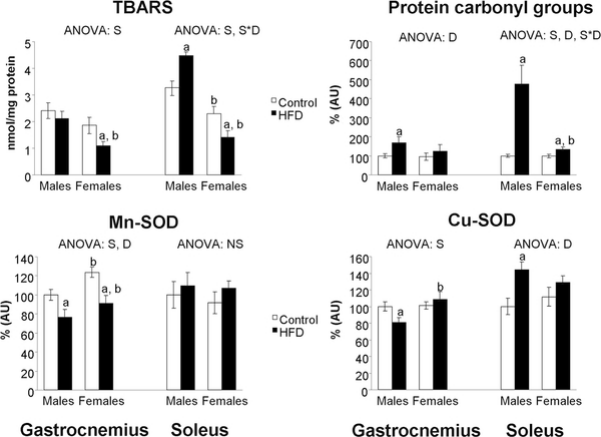
**Skeletal muscle TBARS levels, protein carbonyl groups and Mn-SOD and Cu-SOD protein levels**. TBARS levels are expressed in nmol/mg protein. Protein carbonyl groups, Mn-SOD, Cu-SOD and UCP3 protein levels are expressed as % AU (arbitrary units), where control male rats were set as 100%. Values are expressed as the mean ± S.E.M of eight animals per group. ANOVA (p < 0.05): S sex effect, D HFD effect, S*D sex and HFD interactive effect and NS non significant effect. Student's *t*-test (p < 0.05): ^a^HFD vs control, ^b^female vs male.

**Figure 4:**
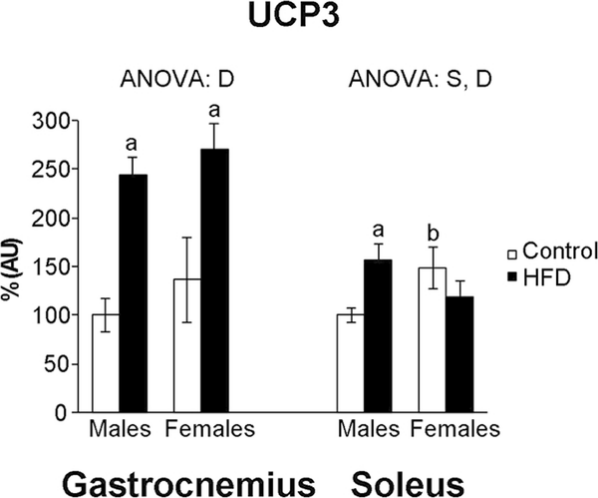
**Skeletal muscle UCP3 protein levels**. UCP3 protein levels are expressed as % AU (arbitrary units), where control male rats were set as 100%. Values are expressed as the mean ± S.E.M of eight animals per group. ANOVA (p < 0.05):S sex effect, D HFD effect.

In gastrocnemius muscle, Mn-SOD (Figure 3) was higher in control female rats than in their male counterparts. No differences between sexes were found in TBARS, protein carbonyl groups, Cu-SOD or UCP3 levels (Figure 4) in control rats. HFD feeding increased UCP3 levels in both sexes and protein carbonyl groups in male rats. In contrast, HFD feeding decreased Mn-SOD protein levels in both sexes - with HFD male rats showing lower levels than their female counterparts - and TBARS levels in female rats.

In soleus muscle, control female rats showed higher UCP3 protein levels and lower TBARS levels than their male counterparts. HFD feeding increased protein carbonyl groups in both sexes, but to a higher degree in male rats (376% vs 35%), and UCP3, Cu-SOD and TBARS levels in male rats. In contrast, HFD feeding decreased TBARS levels in female rats.

## Discussion

HFD feeding induces skeletal muscle mitochondrial biogenesis in both sexes, as the increase of PGC-1α and TFAM protein levels and mtDNA values suggest. Increased levels of PGC-1α, a master regulator of mitochondrial biogenesis [32], would involve the enhancement of skeletal muscle oxidative capacity, whereas mtDNA levels point to an increase of the mitochondrial content. Mitochondrial biogenesis could be understood as an adaptation aimed to counteract the elevated amount of substrate available. The induction by HFD feeding of skeletal muscle oxidative capacity by increasing mitochondrial PGC-1α and respiratory chain units or mitochondria number has been previously reported in male rats [3,33], and is here reported also in female rats. Although the HFD-associated increase of mitochondrial biogenesis is observed in both muscle types, the effect seems more marked in the glycolytic one.

In gastrocnemius muscle, the effect of HFD feeding on mitochondrial biogenesis could be sex-dependent, since male rats, compared to females, show a more patent increase in mtDNA content (80 vs 22%) and PGC-1α (194 vs 71%) and TFAM (204 vs 92%) levels, which only reach statistical significance in the latter. These results suggest a more marked HFD-feeding-induced mitochondrial biogenesis in male rats. Mitochondrial biogenesis is considered a mechanism to counteract the impairment of mitochondrial function that could be consequence of oxidative stress and of the accumulation of toxic lipids, among others [3]. The higher adiposity index that HFD female rats show compared to their male counterparts [34] points to a greater lipid storage capacity of adipose tissue that would protect skeletal muscle from lipid toxic derivates that could impair its function [35]. The lower skeletal muscle TBARS levels shown by HFD female rats compared to males further supports this idea. Thus, the greater adipose tissue expandability of female rats would make the development of strategies to avoid the detrimental effects of lipotoxicity less necessary. In this sense, during evolution, mammalian females have developed mechanisms to handle their energy resources more efficiently than males to facilitate the survival of their progeny and their own [36].

The aforementioned induction of mitochondrial biogenesis in gastrocnemius muscle of both sexes in response to HFD feeding is accompanied by a marked increase of UCP3 levels and could be aimed at compensating the decreased levels of antioxidant enzymes. UCP3 has been proposed to play an important role in the protection of mitochondria against increased ROS production derived from enhanced fat oxidation [37]. Since PGC-1α regulates the expression of UCP3 [32], the increased levels of this coactivator found in obese animals would be aimed, at least in part, to contribute to attenuate oxidative damage in skeletal muscle. A similar effect of HFD feeding on gastrocnemius muscle enhancing UCP3 expression has been previously reported in 6 month-old male and female rats [38] and in 18-month-old female rats, but interestingly not in their male counterparts [26]. Taken together, both the present (performed in 9-month-old rats) and the aforementioned studies suggest the existence of age-dependent sex differences in the capacity to induce UCP3 expression in response to HFD feeding. Our results also suggest that, given the proposed role of UCP3 in the regulation of insulin sensitivity [39], male rats would decrease their capacity to induce gastrocnemius UCP3 expression in response to HFD feeding between 9 and 18 months of age, in accordance with their higher oxidative damage and the earlier impairment of insulin sensitivity that male rats undergo with age compared to females [40].

In soleus muscle, the increase of UCP3 levels in response to HFD feeding turns out to be more attenuated than in the gastrocnemius one, in agreement with previous studies performed only in male rats [41], which showed a higher induction of UCP3 in glycolytic muscles than in oxidative ones. Once more, sex differences in the capacity to induce UCP3 expression are found. The response of male rats to HFD feeding increasing soleus UCP3 levels is accompanied by an increase of Cu-SOD levels that may not be enough to compensate the increase of oxidative stress, as the enhanced oxidative damage indicated. However, in soleus muscle of HFD female rats, the lack of changes in antioxidant enzymes and UCP3 protein levels, as well as in oxidative damage, suggest that UCP3 induction would not be a mechanism to protect soleus muscle from oxidative stress.

All in all, these results point to a more detrimental effect of HFD feeding on both skeletal muscles of male rats, which show a weaker capacity of response in front of an oxidative stress stimulus.

The impairment of insulin sensitivity induced by HFD feeding is also sex-dependent. Thus, although the HFD-induced increase in the levels of insulin resistance markers is more marked in female rats than in males, HFD obese female rats still maintain a better serum profile of insulin sensitivity. This less detrimental profile of obese females is reflected by lower insulin levels and HOMA-IR index values, as well as by the unchanged serum HMW adiponectin to total adiponectin ratio, a marker of the insulin-sensitizing action of this adipokine [18]. The more marked insulin resistance status of HFD male rats is suggested by decreased serum HMW adiponectin levels and HMW adiponectin to total adiponectin ratio values. Moreover, oral glucose tolerance is more altered in HFD male rats [34], which further reinforces this idea.

In spite of the sex dimorphism found in serum adiponectin levels, HFD feeding resulted in an impaired adiponectin response or "adiponectin resistance" in both sexes, as the increase of AdipoR1 protein levels and the decrease of p-AMPK/AMPK ratio indicate. This HFD-associated dysregulation of adiponectin-AMPK signaling has been proposed to contribute to the impairment of insulin sensitivity, through an alteration in fatty acid metabolism that increases lipid accumulation in skeletal muscle which, ultimately, leads to the development of insulin resistance [42-44]. Increased levels of AdipoR1 would reflect a defective compensatory mechanism to overcome this adiponectin resistance, in agreement with previous studies showing a similar response in animal models with features of the metabolic syndrome [42,44].

As regards the involvement of muscle type in the effect of HFD feeding on adiponectin signaling pathway, gastrocnemius muscle shows a more marked response than soleus. In fact, the decreased activation of AMPK in gastrocnemius points to a greater adiponectin resistance than in soleus, which, otherwise, maintains the activation of AMPK unaltered. Given these findings and the above mentioned relationship between adiponectin and insulin sensitivity, our results suggest that gastrocnemius muscle may contribute to the obesity-associated onset of insulin resistance to a greater extent than soleus, despite having a metabolism less dependent on insulin [45].

Although previous studies have reported that adiponectin resistance contributes to the impairment of skeletal muscle oxidative metabolism in HFD fed rodents [42,43], we found that, in response to chronic HFD feeding, adiponectin resistance is accompanied by an enhanced oxidative capacity, which is reflected by an increase of mitochondrial biogenesis. Our results are in agreement with a previous study that reports an increase of mitochondrial content as a consequence of HFD feeding to maintain normal oxidative capacity during later stages of insulin resistance [3]. We suggest that this enhancement of mitochondrial biogenesis may be an adaptation to chronic HFD feeding as an attempt to compensate the deleterious consequences of insulin and adiponectin resistance on skeletal muscle oxidative metabolism.

## Conclusions

In summary, we found that the effects of HFD feeding on skeletal muscle mitochondrial biogenesis could be more enhanced in male rats than in females, which could be attributed to a compensatory response to counteract the more marked increase of oxidative damage associated to HFD feeding in this sex. In contrast, HFD female rats are more protected and maintain a better insulin sensitivity profile than their male counterparts. However, there are no sex differences in the response of skeletal muscle adiponectin signaling pathway to chronic HFD feeding, with both sexes showing a similar profile of adiponectin resistance. The response to HFD feeding is more marked in gastrocnemius muscle and may lead HFD rats to increase mitochondrial biogenesis in order to counteract the deleterious consequences of adiponectin and insulin resistance on skeletal muscle oxidative metabolism. Our results suggest that HFD feeding has a skeletal muscle-type specific effect on adiponectin signaling pathway and a sex-dependent effect on the induction of mitochondrial biogenesis.

## Abbreviations

HFD: High-fat-diet; PGC-1α: Peroxisome proliferator-activated receptor-γ coactivator-1α; TFAM: Mitochondrial transcription factor A; AdipoR1: Adiponectin receptor; AMPK: 5'-AMP-activated protein kinase; UCP3: Uncoupling protein 3; COXII: Cytochrome c oxidase subunit II; COXIV: Cytochrome c oxidase subunit IV; Mn-SOD: Manganese superoxide dismutase; HOMA-IR: Homeostasis Model Assessment of Insulin Resistance; CPT1: Carnitine palmitoyl transferase I.

## Competing interests

The authors declare that they have no competing interests.

## Authors' contributions

YGP has contributed in the conduct of the study, data collection and analysis, data interpretation and manuscript writing. GCA has contributed in the conduct of the study, data collection and analysis. MG has contributed in the design of the study, data interpretation and manuscript writing. IL and AMP have contributed in the design of the study, data collection and analysis, data interpretation and manuscript writing. All authors read and approved the final manuscript.
